# Next-generation composite vesicular systems: an in-depth review of proniosomes in advanced drug delivery

**DOI:** 10.1080/10717544.2026.2614585

**Published:** 2026-01-15

**Authors:** Jagabandhu Bag, Subhankar Mukhopadhyay, Gouranga Nandi, Hein Min Tun

**Affiliations:** a Department of Pharmaceutical Technology, NSHM Knowledge Campus, Kolkata, West Bengal, India; b The Jockey Club School of Public Health and Primary Care, Faculty of Medicine, The Chinese University of Hong Kong, Hong Kong SAR, China; c System Microbiology and Antimicrobial Resistance (SMART) Lab, Li Ka Shing Institute of Health Sciences, Faculty of Medicine, The Chinese University of Hong Kong, Hong Kong SAR, China; d Department of Pharmaceutical Technology, University of North Bengal, Darjeeling, West Bengal, India; e Microbiota I-Center (MagIC), Hong Kong SAR, China

**Keywords:** Proniosomes, composite vesicular systems, niosomes, controlled release, sustainable pharmaceutics, novel drug delivery

## Abstract

Proniosomes represent an advanced composite vesicular platform that integrates non-ionic surfactants, lipids, and biodegradable carriers to significantly improve drug solubility, stability, and transmembrane delivery. These dry powdery formulations are transformed into multiscale niosomes upon contact with a hydrated medium to achieve controlled release and enhanced drug permeability. This seminal review delineates the transformative potential of proniosomal systems in treating various diseases, detailing diverse routes of administration, formulation techniques, mechanisms of action, as well as their advantages and limitations. Proniosomes address major issues such as systemic toxicity, poor solubility, and erratic absorption while maintaining green chemistry principles owing to their biodegradable constituents. By critically analyzing the potential for industrial translation, this review highlights the knowledge gap on clinical studies, scalability, and regulatory issues. The translation potential has even been further enhanced by recent developments in bioconjugation and nanotechnology, such as ligand-anchored proniosomes that enable active targeting. The topic's relevance is evident, as proniosomes complement next-generation biotechnology tools, such as mRNA delivery, while offering a sustainable alternative to liposomes. By compiling the most recent data, this review strives to catalyze innovation in novel drug delivery, making it essential for researchers and pharmaceutical developers.

## GRAPHICAL ABSTRACT

**Figure:**
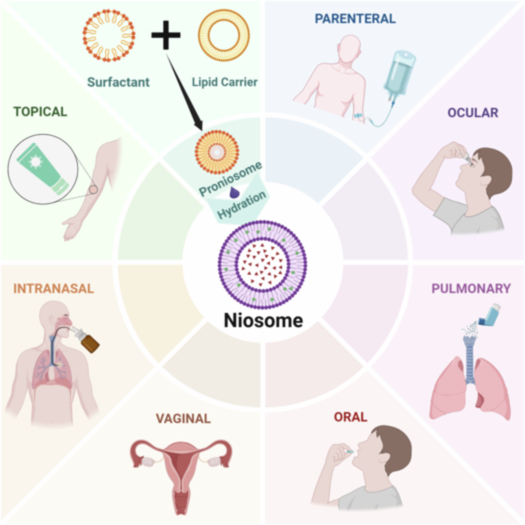

Transformation of proniosome to noisome and their diverse applications in drug delivery. This image was designed with BioRender (https://biorender.com/).

## 1. Introduction

Vesicular drug encapsulation represents a pivotal advancement in targeted therapeutics, optimizing site-specific delivery while minimizing systemic toxicity (Abd El-Alim et al. 2014; Kapoor et al. 2019). This necessity has directed the synthesis of many vesicular drug delivery systems, such as liposomes and niosomes, and provesicular systems, such as proliposomes and proniosomes. Liposomes have colloidal, vesicular structures with an aqueous core and concentric lipidic bilayers that are arranged with various types of surfactants and drugs (Shubhra 2024). Its oral administration is impeded by physicochemical stability issues such as fusion, hydrolysis, and oxidation (Yasam et al. 2014). This catalytic challenge spurred the invention of proliposomes, a fundamental provesicular system designed to address the aqueous stability issues of conventional liposomes (Payne et al. 1986). However, their reliance on phospholipids limits both their cost-effectiveness and biocompatibility. Based on this foundational concept, proniosomes emerged as a superior and more versatile provesicular system by substituting phospholipids with a wider range of tunable non-ionic surfactants (Khatoon et al. 2017).

Proniosomes are dry, freely flowable composite powder products that, when reconstituted with water, form multi-lamellar niosomal structures (Sachan et al. 2021). They have gained popularity as drug transporters and drug-targeting agents since the 1980s to bypass the limitations of liposomes and niosomes (Khatoon et al. 2017). Proniosomes outperform liposomes and niosomes as potential drug carriers because of their greater chemical stability and lower toxicity, along with efficient entrapment of both hydrophobic and hydrophilic drugs (Hu and Rhodes 1999; Khatoon et al. 2017). As a dry product, proniosomes avoid physical stability issues such as leakage, fusion, and aggregation, enhancing their transport, distribution, packaging, and storage (Chen et al. 2019; Shah et al. 2021). A comparative evaluation of vesicular drug delivery systems is detailed in Table 1. While transferosomes and ethosomes offer better skin penetration, proniosomes offer superior overall functionality. Their evolutionary leap from passive dry powdery carriers to stable, stimuli-responsive compositional-functional composite platforms with exceptional versatility across multiple routes (oral, topical, parenteral, pulmonary etc.) position them as next-generation drug delivery vehicles, overcoming the rigidity and shelf-life limitations inherent in conventional liposomes and niosomes. Proniosomes can be readily converted into unit dosage forms, which enormously adds to their utility and potential for large-scale industrial manufacturing (Ahmad et al. 2017).

**Table 1.** Comparative evaluation of vesicular drug delivery systems: proniosomes, niosomes, liposomes, transferosomes, and ethosomes.

Characters	Proniosomes	Niosomes	Liposomes	Transferosomes	Ethosomes	References
**Composition**	Non-ionic surfactants, cholesterol, and carriers	Phospholipids, cholesterol, and non-ionic surfactants	Phospholipids and cholesterol	Phospholipids and edge activators	Phospholipids and ethanol	(Liga et al. 2024; Mishra and Thakur, 2024 ; Watmode et al. 2024; Matharoo et al. 2024; Mahajan et al. 2024)
**Characteristics**	Microscopic and solid colloidal particles	Lipid and microscopic nanoparticles	Microscopic lipid vesicles	Ultra-flexible vesicles	Elastic vesicles	(Liga et al. 2024; Mishra and Thakur, 2024 ; Watmode et al. 2024; Matharoo et al. 2024; Mahajan et al. 2024)
**Flexibility**	Physically stable and rigid	Rigid and elastic	Rigid	High deformability due to surfactant	Elasticity due to ethanol	(Watmode et al. 2024; Matharoo et al. 2024; Yadav et al. 2024; Bhatia et al. 2024)
**Extent of skin penetration**	High permeation	Moderate penetration	Very less penetration	Can easily penetrate	Can easily penetrate	(Kumari et al. 2014; Matharoo et al. 2024; Mahajan et al. 2024; Trucillo et al. 2024; Jaradat et al. 2024)
**Route of administration**	Oral, parenteral, topical, transdermal, vaginal, pulmonary, intranasal, mucosal, ocular	Oral, parenteral, topical, transdermal, pulmonary, intranasal, ocular	Oral, parenteral, topical, transdermal	Topical and transdermal	Topical and transdermal	(Sakdiset et al. 2023; Moammeri et al. 2023; Li et al., 2023 ; Jafari et al. 2023; Matharoo et al. 2024)
**Marketed products**	Ketorolac gel, Risperidone,Losartan potassium, Chlorpheniraminem maleate (CPM)	Minoxidil transdermal gel, Atorvastatin Calcium Nanovesicular gel	Ambisone, Daunoxome	Transferosomes ® (Idea AG)	Nanominox, Decorin cream	(Rajan et al. 2011; Maniyar et al. 2022; Saxena et al. 2023; Izhar et al. 2023)

This review provides an overview of current knowledge on proniosomes by critically dissecting their identity as engineered composite systems and elucidating how strategic integration of polymers, ligands, and other functional excipients transforms them beyond mere niosome precursors. We also provide a thorough scholarly resource by combining mechanisms, formulation techniques, administration routes, and therapeutic uses. Furthermore, we address translational hurdles by assessing the clinical trials, the patent environment, regulatory concerns, and ongoing restrictions to develop a strategic roadmap for realizing proniosomes as transformative, cutting-edge drug delivery tool.

## 2. Mechanistic insights into proniosomal drug delivery

**Figure 1.**
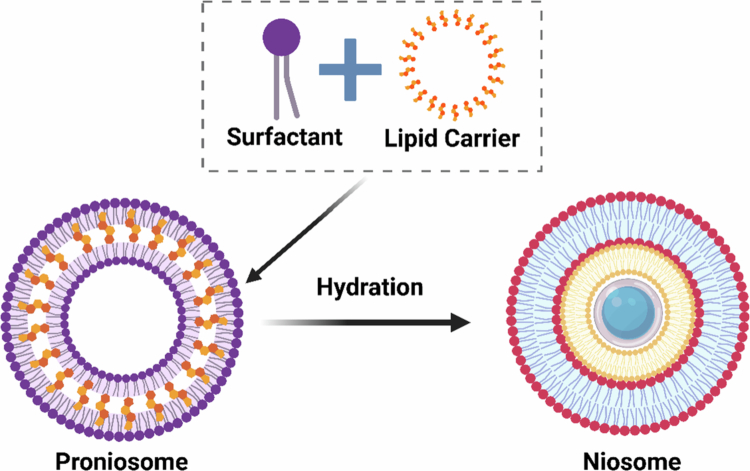
Mechanism of proniosomes behind drug delivery. This image was created with BioRender (https://biorender.com/).

Proniosomes are the inactive form of niosomes; after hydration, they transform to the active niosomal form. There are two ways of hydration: first, through direct moisture uptake by the skin, and second, in the presence of solvents such as water and buffer solutions. These composite biomaterials encapsulate the drug in their hierarchical structure, shielding it from external conditions and enabling controlled release triggered by environmental factors such as temperature or pH. They can also transport drugs to specific sites in the body, such as cancer cells and inflamed tissues (Khatoon et al. 2017; Shehata et al. 2021). Figure 1 illustrates the drug delivery mechanism of proniosomes, including their hydration-triggered conversion to niosomes.

Key physicochemical parameters, primarily the surfactant's chemical structure and hydrophilic‒lipophilic balance (HLB), control the effective transformation of proniosomes into multiscale functional niosomes (Kumar and Rajeshwarrao 2011; Ahmed et al. 2025). Additionally, the molar ratio of surfactant to cholesterol significantly influences bilayer integrity and stability. The hydration conditions are equally critical, as the process must exceed the surfactant's phase transition temperature (Tc) to enable sufficient molecular mobility for spontaneous self-assembly into vesicles (Kumar and Rajeshwarrao 2011). The vesicle size, polydispersity, and drug entrapment efficiency are directly governed by hydration parameters, including the agitation method, duration, and aqueous medium properties, such as pH, ionic strength, etc. (Alsarra et al. 2005). Stable and effective proniosomal-turned niosomal dispersions require careful regulation of these factors. Table 2 presents proniosomes as a revolutionary platform that exhibits unmatched adaptability for a wide range of medicinal uses, from transdermal antipsychotics to oral antibiotics. Improved medication penetration, prolonged release, and decreased toxicity are made possible by their special compositions (surfactant/cholesterol matrices). Notably, proniosomes are the next frontier in precision nanomedicine since they outperform traditional administration in terms of bioavailability (such as atenolol's 365-fold increase in area under the curve (AUC) and targeted action (ocular pressure management).

**Table 2.** Systematic compilation of proniosomal formulations detailing their therapeutic applications, compositions, and formulation advantages.

Drug	Purpose of administration	Route of administration	Composition	Advantages	References
Cephradrine	Respiratory infections	Oral	Sorbitol/maltodextrin/span 60/cholesterol	Enhanced antibacterial effect with controlled release property.	Saim et al. (2022)
Flurbiprofen	Osteoarthritis	Oral	Brij 35/cholesterol	Increase in percutaneous permeation.	Kumar and Jain, 2016
Fluticasone propionate	Corticosteroid	Oral	Xanthum gum/sodium alginate	Reduced systemic adverse effects and prolonged duration of action.	Ahmed et al. (2021)
Itraconazole	Antifungal	Oral	Mannitol/maltodextrin	Reduced side effects.	Ismail and Khattab (2018)
Tizanidine Hydrochloride	Anti-spastic	Oral	Cholesterol/span 40/span 60	Enhancement of anti-nociceptive effect.	Mohsen et al. (2023)
Fluconazole	Antifungal	Oral	Span20/soya lecithin/ethanol	Maximum drug release and permeability.	Salam et al. (2021)
Aceclofenac	Pain and inflammation	Oral	Span 60/orabase gel	Enhanced permeation and prolonged drug release for oro-dental conditions.	Khindri et al. (2015)
Letrozole	Aromatase inhibitor	Parenteral	Sorbital/sucrose/ethanol	Enhanced drug dissolution.	Khudair et al. (2020)
Flurbiprofen	Osteoarthritis	Parenteral	Span 80: Span 20/sorbitol/cholesterol	long-lasting anti-inflammatory effect and a decrease in dosage frequency.	Kumar and Jain, 2016
Levoflaxacin	Reduce bacterial infections	Dermal	Span 20/lecithin/cholesterol	Better drug entrapment efficiency.	Salam et al. (2021)
5-Fluorouracil	Antimetabolite drug	Dermal	Soya lecithin/span 20	Penetration enhancement.	Jampala and Gadela (2021)
Curcumin	Anti-oxidative and anti-inflammatory	Dermal	Cholesterol/surfactants	Greater penetration and effectiveness.	Akbari et al. (2020)
Rutin	Reduce arthritis pain	Dermal	Span 60/cholesterol/soy lecithin/ethanol	Reduced toxic effects.	Pinzaru et al. (2021)
Cilostazol	Reduce leg pain	Transdermal	Span 60/cholesterol	Potential delivery across the skin.	Nemr et al. (2021)
Lornoxicam	Analgesic and anti-inflammatory	Transdermal	Lutrol F68/cholesterol/lecithin	Better efficacy.	Vijaya et al. (2018)
Risperidone	Schizophrenia	Transdermal	Surfactants/soya lecithin/cholesterol	Improvement in the mean residence time ensures prolonged blood circulation.	Sambhakar et al. (2017)
Atenolol	Treat hypertension	Transdermal	Soya lecithin/ethanol	Increased percentage of AUC by 365.38-fold.	Ramkanth et al. (2018)
Tramadol	Severe pain	Transdermal	Soya lecithin/cholesterol/HPMC	Improvement of antinociceptive and anti-inflammatory effects.	Shah et al. (2020)
Brimonidine tartrate	Reduction of intraocular pressure	Ocular	L-a-phosphatidylcholine/cholesterol/brij 52	Enhancement of drug release rate and greater bioavailability.	Emad Eldeeb et al. (2019)
Dorzolamide-HCl	Intraocular pressure reduction	Ocular	L-a-lecithin/span 40/cholesterol	Reduce intraocular pressure more effectively than normal formulations.	Fouda et al. (2018)
Voriconazole	Fungal or yeast infections	Ocular	HPMC/carbopol 940	Sustained release property.	El-Emam et al. (2020)
Sildenafil/Vardenafil/Tadalafil	Erectile dysfunction	Topical penile therapy	Span/lecithin/cholesterol	Enhancement of sexual activities.	Mohamed et al. (2023)
Vismodegib	Metastatic basal cell carcinoma	Pulmonary	Span/lactose/mannitol	Better drug deposition, cytotoxic activity, and effective bioavailability.	Gamal et al. (2020)
Gemifloxacin	Pneumonia	Pulmonary	Span 60/tween 60/poloxamer 407/brij 58/cholesterol PB	High antibacterial activity.	El-Emam et al. (2023)

## 3. The architectural blueprint of proniosomes: types and composite nature

Proniosomes' formulation design directly contributes to their structural and functional versatility. A deeper understanding arises from recognizing their inherent identity as constructed composite systems, although they can be conventionally classified according to their physical appearance and the size of the vesicles they generate upon hydration. Their capacity for sophisticated functional assembly, as well as their structural makeup, gives them a multifaceted composite character.

### 3.1. Conventional classification based on vesicular morphology

Proniosomes are traditionally classed based on the morphology of the niosomes generated during hydration, which is ultimately regulated by formulation composition and processing settings (Kakar et al. 2010; Khindri et al. 2015; Reddy and Gandla 2022). Small unilamellar proniosomes (SUPs) are dry, free-flowing particles containing non-ionic surfactants such as Span 60 or Tween 80, cholesterol, and other stabilizers, with a size range between 25 and 100 nm. Sonication or microfluidization methods are mostly used to develop SUPs that promote high drug encapsulation efficiency, controlled release, and enhanced therapeutic efficacy. Large unilamellar proniosomes (LUPs; size 100–1000 nm) are dry powder composition prepared of non-ionic surfactants, cholesterol, and other additives. LUPs are developed using ether injection or reversed-phase evaporation, resulting in vesicles with a large aqueous core ideal for hydrophilic payloads and a lipid bilayer that can hold hydrophobic drugs. Dry granular proniosomes (DGPs) are a widely investigated class of proniosomal systems. These free-flowing powders are composed of non-ionic surfactants, cholesterol, and other excipients. They are typically produced by freeze-drying, which removes the water content and leaves a dry, granular material. Upon simple hydration, the amphiphilic block copolymers spontaneously organize into vesicular structures with a lipid bilayer arrangement. The hydrophilic drugs are then encapsulated in the aqueous core and the hydrophobic drugs are entrapped in the lipid layers. DGPs have longer shelf-life than liquid proniosomes. Dry granular proniosomes are of two types:

***Sorbitol-based proniosomes:*** Sorbitol-based proniosomes are stable, efficient, and versatile drug delivery systems, with sorbitol serving as a key excipient to stabilize this vesicle. This system offers the advantages of improved stability, easy storage, and controlled drug release upon rehydration.

***Maltodextrin-based proniosomes:*** These are one of the specific types of dry powders that employ maltodextrin polysaccharide along with non-ionic surfactants, cholesterol, and stabilizers. Proniosomes require a filler, which is maltodextrin in this case, to keep the powdery proniosomes intact when stored and offer superior characteristics, such as greater stability and integrated drug release.

### 3.2. Proniosomes as engineered composite delivery systems

A proniosome is a solid-phase composite matrix in which the active pharmaceutical ingredient is distributed inside a precisely tailored blend of components (e.g. surfactant, lipid, and carrier matrices), each of which plays an important role in determining the performance of the final system (Khatoon et al. 2017). In addition to their intrinsic structural and compositional complexity, the next-generation potential of proniosomes is unlocked through advanced functional compositing, which involves strategically engineered integrations that evolve them from simple drug reservoirs to active and adaptive delivery platforms. This transformation is driven by ligand-anchored targeting, where the surface functionalization of derived niosomes with targeting ligands (e.g. folic acid, peptides, and antibodies) creates drug carrier-targeting triads, enabling active drug delivery, enhanced therapeutic efficacy, and a significant reduction in off-target toxicity. Further sophistication is achieved by polymer-hybrid engineering, where the integration of biopolymers such as chitosan (Khatoon et al. 2019), carbopol (Mohmed and Ahmed 2016), hydroxypropyl methylcellulose (Shah et al. 2020), or maltodextrin (Sahoo et al. 2014) yield mucoadhesive hybrid vesicles with prolonged mucosal retention at mucosal surfaces, or by stimuli-responsive ‘smart’ composites that trigger payload release via pH, temperature, or redox cues at pathological sites (Khatoon et al. 2017; Darson et al. 2023). Emerging research should focus on developing hybrid lipid‒polymer proniosomes that synergize lipid biocompatibility with polymeric mechanical resilience and controlled release kinetics to create systems with exceptional robustness and tunability (Gajbhiye et al. 2023). These engineered composite strategies endorse their position as a next-generation system, providing a varied and powerful toolkit to address the various issues of current drug delivery.

## 4. Fabrication strategies: from conventional techniques to next-generation manufacturing

**Figure 2.**
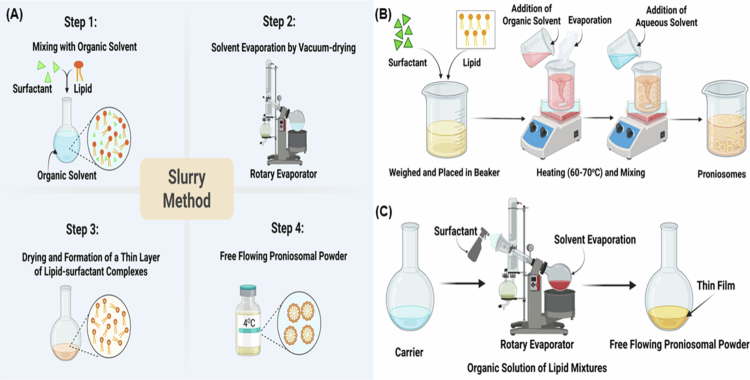
Schematic representation summarizing the fundamental preparation processes for proniosome production: (A) slurry method, (B) coacervation phase separation technique, and (C) spray coating method. This image was generated with BioRender (https://biorender.com/).

The fabrication of proniosomes relies on a series of well-established engineering techniques that convert surfactants, lipids, and carrier matrices into stable, free-flowing dry powders. Conventional approaches (as illustrated in Figure 2) are well established and offer a fundamental understanding of proniosome biofabrication. The slurry method utilizes a flask with a circular base to make the slurry with a particular volume of organic solvent, lipid carriers, and surfactants, followed by vacuum-drying to form free-flowing proniosomal powder. The obtained product needs to be placed in a sealed container in a refrigerator at 4 °C to minimize contamination and product degradation (Khudair et al. 2020; Shruthi et al. 2020). Coacervation phase separation is another widely applicable technique for the preparation of proniosomes. In this method, weighed ingredients are placed into a wide-mouthed glass beaker with surfactants and lipid carriers, followed by the addition of an organic solvent. Next, the ingredients are well mixed and heated over a water bath at 60–70 °C until the surfactants are dissolved properly. During the process, precautions must be taken to prevent solvent loss due to evaporation. The mixture should remain in a water bath at a steady temperature while the aqueous phase is added. In the end, the resulting solution is cooled to form proniosomes (Emad Eldeeb et al. 2019; Tareen et al. 2020; Aboumanei and Mahmoud 2020). Furthermore, the spray-coating method involves spraying an organic solvent-based surfactant solution over carrier/coating materials, followed by solvent evaporation to develop a proniosomal formulation. The surfactant creates a thin layer on the carrier materials, which is then hydrated and forms multi-lamellar vesicles (Radha et al. 2013; Mittal et al. 2020).

While traditional methods remain the foundation of laboratory-scale proniosomal development, next-generation fabrication approaches promise greater precision, scalability, reproducibility, and environmental sustainability. For instance, adopting microfluidics technology to rapidly mix precursors at the microscale provides superior control over vesicle size and dispersity (Shepherd et al. 2021; Obeid et al. 2022). Meanwhile, supercritical fluid (SCF) technology, which primarily uses carbon dioxide (CO_2_) as a SCF, presents a potential, solvent-free alternative that overcomes the challenges of residual organic solvents, thereby simplifying downstream processing and improving safety profiles (Falconer et al. 2015; William et al. 2020). Integrating a more holistic quality-by-design (QbD) approach and high-throughput screening will also be crucial, allowing for the systematic optimization of critical formulation and process parameters (Zagalo et al. 2022). Ultimately, this data-driven method is pivotal for developing industrially viable and regulatory-compliant technologies that will elevate proniosomes from a potential breakthrough to a therapeutically translatable, next-generation platform.

## 5. Physicochemical profiling of proniosomes

Extensive physicochemical characterizations are required to ascertain the quality and forecast the in-vivo effectiveness of niosomes derived from proniosomes. After hydration, the resulting vesicles are subjected to analysis to observe vesicle morphology, size, and lamellarity using scanning electron microscopy (SEM) and transmission electron microscopy (TEM) (Verma et al. 2016). Dynamic light scattering (DLS), which yields vital information on vesicle homogeneity and colloidal stability, is used to quantify particle size, polydispersity index (PDI), and zeta potential (Shruthi et al. 2021). The entrapment efficiency (%EE), a critical performance parameter, is measured to determine the quantity of drug that is successfully encapsulated. This is typically accomplished by centrifuging the unentrapped drug and analyzing the supernatant (Kapil et al. 2012). In addition, in-vitro drug release profile can be evaluated by using various techniques, such as the dialysis method, to determine the drug release kinetics (Verma et al. 2016). To examine drug‒excipient interactions and validate the amorphous dispersion of the drug within the carrier, solid-state characterization of the dry proniosomes powder is also carried out using methods such as differential scanning calorimetry (DSC) and X-ray diffraction (X-RD). The funnel and cylinder methods are used to measure the angle of repose (θ) (Mishra et al. 2011). Proniosomal powder flows from a height of 10  cm to create a cone in both directions, and the angle is determined using the formula:

$$\theta =Tan^{-1}\;\times \;\frac{h}{r}$$

Here, ‘*r*’ is the base radius and ‘*h*’ is the cone height.

In addition, the safety and storage stability of the formulation are also evaluated (Verma et al. 2016; Ramkanth et al. 2018). Collectively, these studies establish a robust physicochemical profile, ensuring that the formulation is suitable for its intended role as a drug delivery system.

## 6. Proniosomes: a versatile drug delivery platform

Proniosomes are flexible drug delivery systems that can be administered in a variety of ways. Their particular uses in the controlled and targeted delivery of drugs will now be covered in depth in the section below.

### 6.1. Oral delivery

Oral drug delivery has been the prime mode of drug administration since the beginning of the medical era (Jain 2008). However, it has been associated with several pitfalls. Drugs that are unstable in the gastrointestinal (GI) tract, undergo degradation by enzymatic or acidic reactions, exhibit minimal permeability through the intestinal epithelium, or undergo extensive first-pass metabolism must be delivered in special dosage forms (Kaur et al. 2019). To negotiate the gastro-intestinal barrier, drugs need to be present in a dissolved form (Florence and Attwood 1998). This requirement gives rise to a variety of nanocarriers, such as liposomes (Caddeo et al. 2017), niosomes (Khan et al. 2016), lipid nanoparticles (Kraft et al. 2014), micelles (Chen et al. 2013), gold nanoparticles (Chen et al. 2014), quantum dots (Chen et al. 2014), and proniosomes (Mittal et al. 2020). A number of investigations show that oral proniosomal powders can improve the solubility and bioavailability of poorly soluble medications (Nasr 2010). Song et al. developed a free-flowing and stable proniosomal formulation of vinpocetine, a neurological drug with poor solubility, to improve its oral bioavailability and GI absorption. According to the results, vinpocetine-loaded proniosomes showed higher (up to 4.9-fold) bioavailability compared to the free drug (Song et al. 2015). The authors opined that, on reaching the GI lumen, proniosomes are transformed into niosomes by hydration, which is followed by endocytosis. Second, niosomes enter the lymphatic system, which helps them to avoid the first-pass effect and improves their bioavailability. In another study, nateglinide, an anti-diabetic medicine, was encapsulated in proniosomes to increase its oral bioavailability and absorption. They reported significantly higher (32%) plasma concentrations of nateglinide in rabbits compared to the pure drug (Sahoo et al. 2014). Similar to these findings, in a rat pharmacokinetic study, an optimized proniosomal formulation enhanced the oral bioavailability of lipophilic irbesartan by 2-fold over a pure drug suspension (Mujtaba et al. 2024).

These findings established proniosomes’ superiority in oral delivery of poorly hydrophilic drugs compared to others (Khatoon et al. 2017). Another group developed a proniosomal carrier system utilizing various types of molecules, including spans, cholesterol, and maltodextrin (Gad et al. 2014). The system was used to entrap a combination of two drugs, metronidazole and doxycycline hydrochloride. Both medicines were successfully incorporated into the proniosomal structure without any chemical interactions with other substances. The proniosomal powder remained stable for 3 months at 2–8 °C and exhibited free-flowing characteristics. The dry powder was easy to handle, permitted accurate dosing, and resulted in better patient compliance (Gad et al. 2014). While the preparation of proniosomes is easy and has the potential to positively impact drug delivery, there is concern about their large-scale manufacturing (Yuksel et al. 2016). During mass production, the intricate arrangement of the drug and excipient systems can be completely changed and may revert to conventional dosage forms. Hence, the preparation of oral proniosomal tablets should require proper coating, which might increase production costs (Khatoon et al. 2017).

### 6.2. Parenteral delivery

Drug administration through the parenteral route is a generic strategy typically used for pharmacological components with a restricted therapeutic index and low bioavailability. It is also useful for patients who suffer from swallowing problems, delayed gastric emptying, intestinal motility, vomiting, or are unconscious (Nkanga et al. 2020). In emergencies, when the oral route is not recommended for practical considerations, it is used to provide instant relief. However, this almost ideal system also has several significant drawbacks, including the need for skilled manpower, a rapid decrease in systemic concentration, and frequent administration. One of these drawbacks can be overcome by using biodegradable polymeric nanosystems, which can sustain drug release for an extended period and help in reducing the frequency of dosing and minimizing hazardous side effects (Kim et al. 2023; Din et al. 2017a; Din et al. 2017b).

Sterilization is an essential requirement for parenteral dosage forms. Though a variety of methods are available for these, each has drawbacks of its own. The use of cryoprotectants during freeze drying and sterilization by gamma-irradiation can cause significant physiological changes, especially in terms of particle size and drug release rate (Bozdag et al. 2005). However, proniosomal formulations are devoid of these problems. They can be quickly sterilized, reconstituted with a little agitation, and maintained without any changes in particle size or drug release rate (Nasr 2010). Moreover, proniosomes can be easily divided into unit dosage forms for parenteral administration and remain stable during storage and transportation (Verma et al. 2016). A recent study has shown that the anti-inflammatory properties of flubiprofen can be improved by formulating them into proniosomes. The formulation could maintain the systemic concentration of the drug for a prolonged period of time (3 days), which reduced its frequency of dosing (Verma et al. 2016). In short, proniosomes are stable, accessible, beneficial, and promising advanced substitute for current vesicular and colloidal drug delivery systems with the potential to transform parenteral drug delivery.

### 6.3. Topical and transdermal delivery

Human skin, a selective barrier that prevents the penetration of unwanted substances while maintaining hydration, has emerged as a promising route for systemic drug delivery (Fukushima et al. 2011). The route has many benefits, among which non-invasiveness and avoiding first-pass metabolism are prime. Drugs prone to GI degradation or difficult to regulate a steady-state plasma concentration are ideal candidates for this delivery route (Kováčik et al. 2020; Ramadon et al. 2022). However, limited drug permeation through the complex structure of the stratum corneum remains an important backdrop. Over the past decade, studies have shown that vesicular drug delivery systems effectively overcome shortcomings in dermal and transdermal administration (Chacko et al. 2020; Witika et al. 2021). There are many types of vesicular systems investigated for topical and transdermal administration, including liposomes, noisomes, and proniosomes (Muzzalupo 2016). Among these, proniosomes offer a unique advantage because of their adherence to the stratum corneum, convert into niosomes when moistened, and subsequently increase skin penetration through the stratum corneum (Shah et al. 2021). These vesicular carriers serve as drug reservoirs in addition to their tunable surface drug release properties (Kamel et al. 2013).

Numerous studies have been carried out to optimize the composition of the constituent molecules of pronisomes (non-ionic surfactants, cholesterol, phospholipids, etc.) that favor the permeation of drugs through the complex structure of the skin. An experiment that evaluated the effectiveness of drug penetration and entrapment of flubiprofen in proniosomes has shown that the optimized batch (maximum amount of nonionic surfactant and minimum amount of cholesterol) of proniosomes had high drug entrapment (39.45%) efficiency and skin permeability (Zidan and Mokhtar 2011). Mehta et al. reported that boswellic acid, a poor bioavailability drug, has improved its absorption (98.52%), release rate (84.83 ± 0.153 mg/cm^2^ in 24 h), and bioavailability when loaded in a proniosomal gel. In a compositional study, boswellic acid-loaded proniosomal gels containing leukotriene inhibitors showed remarkable (*p* <0.001) anti-inflammatory properties compared with the marketed voveran gels (Mehta et al. 2016). Similarly, in the treatment of overactive bladder, transdermal administration of tolterodine tartrate proniosomes resulted in better therapeutic efficacy and high drug permeability (35%–42%), while their oral formulation showed many adverse effects (Rajabalaya et al. 2016). An added advantage of transdermal proniosomes is that they can be used in patches, films, and gel carriers to provide bioadhesive properties to improve skin contact using cost-effective raw materials (Khatoon et al. 2017).

### 6.4. Ocular delivery

In general, low bioavailability is a common issue with ocular dosage forms due to the blinking reflex, low eye capacity, nasolacrimal drainage, and resistance offered by the corneal and conjunctival epithelial barriers. These factors reduce the retention time of drug molecules in the eyes, leading to their limited absorption (Kaur et al. 2012). Therefore, in order to achieve the desired level of therapeutic effectiveness, conventional eye formulations need to be deposited multiple times (Luo et al. 2011), which often leads to patient noncompliance. Several novel ocular drug delivery systems, such as in-situ gels (Gupta et al. 2015), liposomes (Dai et al. 2013), in-situ nanosuspensions (Luschmann et al. 2013), nanoparticles (Nagarwal et al. 2012), nanoemulsions (Garg et al. 2013), and proniosomes (Abdelkader et al. 2012) have been developed to overcome this difficulty. Among these, proniosomes are believed to be most suitable for ocular delivery. Ketoconazole-loaded proniosomal gel increased the bioavailability (up to 20-fold) of the drug in the eyes by prolonging their contact time in the corneal cavity, providing prolonged and sustained action at specific sites and preventing enzymatic metabolism by tears (Abdelbary et al. 2017). This finding aligns with the study by Li et al., in which they produced a tacrolimus-loaded proniosomal formulation. The developed formulation was targeted into rabbit’s cornea, where proniosomes were converted into niosomes in the presence of an aqueous phase and showed better permeability and greater retention time compared to conventional ointments (Li et al. 2014). In a corneal xenograft transplantation model using Sprague-Dawley rats, tacrolimus-loaded proniosomes significantly retarded corneal allograft rejection and sustained retention time (13.86 ± 0.80 days) compared to cyclosporine eye drops (Li et al. 2014).

### 6.5. Vaginal delivery

Traditionally, the vaginal route is used to treat local microbial infections, mainly vulvovaginal candidiasis and bacterial vaginosis (Lin et al. 2021). Recently, its potential as a drug delivery port for other medicines, such as antimicrobials, antimycotics, sex hormones, and peptides, has been recognized (Ensign et al. 2014). Vagina has a unique set of pearls and perils. On the plus side, it has a rich blood supply, which drains into the inferior vena cava through the iliac veins, bypassing first-pass metabolism as well as good permeability. On the negative side, it has significant idiosyncrasies that result in variances in the bioavailability of many drugs. Several physiological and non-physiological factors, such as menstruation or menopause, sexual activity, and personal cleanliness, may alter the bioavailability of vaginally administered drugs (Vanić and Škalko-Basnet 2013; Yang et al. 2013). The natural clearing process through vaginal mucus secretion also limits many drug molecules from their proper administration at the specific site of action (das Neves et al. 2011). The bioavailability of the vaginal systems is often compromised by leakage and the short residence time of the dosage form. Hence, the focus has shifted to the development of novel formulations with good retention, release, and diffusion properties. Different micro and nano innovative delivery techniques, such as hydrogel (Khade et al. 2014), vaginal sponges (Furst et al. 2015), nanoparticles (Meng et al. 2011), nanocapsules (Santos 2013), solid‒lipid nanoparticles (Alukda et al. 2011), niosomes (Ning et al. 2005), and liposomes (Palac et al. 2014), have been investigated. Many of these studies focused on exploiting mucoadhesive properties, as they play a crucial role in keeping the dosage form in close contact with the vaginal mucosa (de Araújo Pereira and Bruschi 2012). Among vaginal dosage forms, including tablets, solutions, foams, suppositories, and inserts, semisolid dosage forms, particularly gels enjoy the maximum popularity (Neves et al. 2014). Proniosomal gel systems, owing to their strong mucoadhesive properties, hold significant potential as effective carriers for vaginal drug administration. Abdou et al. developed proniosomes of the antifungal drug terconazole to treat vaginitis (Mohmed and Ahmed 2016). It is converted to niosomes upon contact with the vaginal aqueous medium. Microbiological assessment indicated the prolongation of residence time (3 h) of the drug in the vagina, leading to better therapeutic outcomes (Mohmed and Ahmed 2016).

### 6.6. Pulmonary delivery

The lungs have garnered attention as a drug delivery route, given their role as the primary organs affected by various serious respiratory diseases (Misra et al. 2011). Its importance has recently increased manifold as it has been recognized as an excellent gateway for the systemic administration of drugs. With an enormous surface area (>100 m^2^) and thin epithelial layers specifically designed for gaseous exchange, the lungs can absorb drug molecules more effectively than most other absorption sites (Nasr 2010). The double blood supply of the lungs is also a significant advantage. Pulmonary-delivered drugs enter systemic circulation directly via the pulmonary veins into the left atrium, completely bypassing first-pass hepatic metabolism. The application of nanotechnology, as well as portable inhalers, makes this method even more convenient for patients (Zhou et al. 2014; Kuzmov and Minko 2015). As a result, for both local and systemic effects, the lungs can be used as the most effective drug delivery route.

Proniosomal drug delivery systems with various types of nasal nebulizers were successfully developed by Elhissi et al. (2013). Beclomethasonedipropionate (BDP) was used as a model drug. Following pulmonary delivery, proniosomes were converted into niosomes, exposing the intrinsic characteristics of aerosols. The findings revealed that proniosomes had a greater level of BDP entrapment (35.4%) than conventional niosomal dosage (27.5%) forms. Additionally, the developed aerosols from the nebulizer systems were able to yield large amounts of drug molecules with a high fine particle fraction (FPF) (Elhissi et al. 2013). In a different study, Abd-Elbary et al. used the nonionic, biocompatible, and biodegradable surfactant ‘sucrose stearate’ to design a controlled-release nebulizable delivery system for cromolyn sodium-loaded proniosomes. The findings demonstrated that proniosomes quickly hydrated (within 2 min) and effectively absorbed through the pulmonary circulation and showed better therapeutic efficacy (Abd-Elbary et al. 2008).

### 6.7. Intranasal delivery

Drug delivery through the intranasal route is a potentially non-invasive technique to avoid the blood‒brain barrier (BBB) and deliver medicines directly to the central nervous system (CNS). The nasal mucosa, with its olfactory receptor neurons, trigeminal nasal pathways, nasal lymphatic, and porous endothelium, can directly transport the drugs to the CNS (Bors and Erdő 2019). Many therapeutic components are administered to the CNS through the intranasal route (Misra et al. 2016). Moreover, this route allows self-administration and is cost-effective in nature (Bhatt et al. 2015). Despite its advantages, the nasal route presents limitations, including enzymatic degradation, mucosal irritation, potential tissue damage from chronic use, and rapid mucociliary clearance, which collectively constrain its therapeutic utility (Ali et al. 2010). Drug delivery through nanosized carriers (1–100 nm) that can easily cross the brain endothelium membrane can minimize these drawbacks. Nanocarriers can also prevent the enzymatic breakdown of drug molecules and prevent them from harsh environments, improving their bioavailability and cellular absorption (Alsarra et al. 2010; Gurrapu et al. 2012). Proniosomes are promising vesicular drug delivery systems that can deliver both small and large therapeutic agents effectively through the intranasal route to the CNS (Alsarra et al. 2010). Khatoon et al. showed that the unique vesicular structure of proniosomal gels enhanced duloxetine absorption. Their ex-vivo and in-vivo studies have demonstrated that a mucoadhesive proniosomal gel of an antidepressant drug, duloxetine, for intranasal delivery offers promising effects, with extended intranasal retention (54% release over 8 h), enhancing bioavailability and significantly (*p* <0.005) improving transmucosal permeation (Khatoon et al. 2019).

## 7. Therapeutic applications of proniosomes

Proniosomes have been effectively used in a variety of therapeutic domains by harnessing their special delivery benefits. The following section will detail their specific applications, including cardiac, diabetic, and hormonal therapy, as well as in gene, peptide, and hemoglobin delivery.

### 7.1. Application in cardiology

In cardiology, proniosomal drug delivery systems present a potential strategy, especially for improving the bioavailability of drugs such as statins. This technique increases the solubility and stability of pharmaceuticals by encasing them in a proniosomal formulation, which becomes niosomes upon hydration, such as atorvastatin, a medicine based on this mechanism, which is used to prevent heart attacks and strokes. Better absorption and controlled release may result from this tailored distribution, which will ultimately reduces adverse effects and increases patient compliance. Proniosome utilization is a noteworthy development in the efficient treatment of cardiovascular illnesses (Eltellawy et al. 2021). Many studies have shown that the proniosomal system prolongs entrapment efficiency and drug release time into the body. To this end, transdermal delivery of captopril-loaded proniosomes has achieved high encapsulation efficiency (66.7%–68.7%) and sustained release kinetics in hypertensive models (Gupta et al. 2007). Lisinopril dihydrate, an angiotensin-converting enzyme (ACE) inhibitor, is another example of a proniosomal drug delivery used in cardiology. Lisinopril was encapsulated in a proniosomal gel comprising cholesterol and non-ionic surfactants, which allowed the medication to hydrolyze and form niosomes (Ahmad et al. 2011). This method improves the drug's solubility and stability, which helps to enhance GI tract absorption effectively. A controlled release of lisinopril sustained therapeutic levels over an extended period of time (24 h) and improved blood pressure and minimized adverse effects (Ahmad et al. 2011).

### 7.2. Application in diabetes

Drug delivery based on this unique strategy shows several advantages for the management of diabetes. The glibenclamide proniosomal formulation enhanced the solubility of the drug and represents better oral bioavailability. A study assessed that blood glucose levels decreased by approximately 73%, and the formulation was stable compared to other conventional dosage forms (Alshora et al. 2023). Another study established some valuable results for amitriptyline and liraglutide-containing proniosomal formulations to control diabetic neuropathy. It significantly controlled blood glucose level and reduced neuropathic pain, oxidative stress, and inflammation as compared to the oral and subcutaneous administration of amitriptyline and liraglutide. The therapeutic efficacy was greater with minimizing side effects (Eissa et al. 2023). Metformin is another example of proniosomal drug delivery in diabetes. This novel formulation plays a vital role in minimizing GI side effects, increasing solubility and bioavailability, and enabling regulated release, which eventually improves patient adherence in diabetic drug delivery (Loona et al. 2012). Sahoo et al. performed another study on maltodextrin-based proniosomes of nateglinide to reveal their bioavailability in a controlled manner by using a rabbit model. In their study, an HPLC method with photodiode array detection was developed to validate the nateglinide concentration in plasma. As per the findings, rapid release was converted to controlled release with a good retention time (8.72 min), and showed more effective bioavailability (Sahoo et al. 2014).

On the other hand, glibenclamide-loaded proniosomal gel was developed to protect testicular tissue from hyperglycemia fluctuation and ROS. These results indicate that this formulation could reduce oxidative stress in testicular tissues by upregulating anti-ROS enzymes and increasing insulin production (Alyami et al. 2024).

### 7.3. Application in hormonal therapy

Over the past few decades, various studies have highlighted the current value of proniosomes in hormonal therapy for managing both serious and non-serious health conditions in the human body. Proniosome-based levonorgestrel therapy was introduced for in-vitro and in-vivo assessment to assess its efficacy in emergency medical conditions. These findings discovered a higher entrapment efficiency of the drug, which showed better permeability, good therapeutic effectiveness, with minimized side effects (Vora et al. 1998). Another study demonstrated by development and evaluation of letrozole-based proniosomes. They reported that the formulation had controlled drug release and increased drug entrapment efficiency (~74%) with an effective therapeutic index compared to other dosage form for the treatment of breast cancer (Khudair et al. 2020).

A formulation of proniosomal estradiol showed improved transdermal delivery, near-quantitative drug encapsulation (~100%), and increased bioavailability post hydration. A significant improvement over conventional delivery techniques, this vesicular system allows for regulated hormone release, reduces systemic side effects and maximizes therapeutic efficacy for hormone replacement therapy (Fang et al. 2001).

### 7.4. Application in peptide drug delivery

The proniosomal drug delivery technology addresses major issues in oral peptide delivery by shielding proteins and peptides from enzymatic degradation. The peptides are saved from GI degradation using a proniosomal dosage form, as the vesicles are capable of providing a surface coating on drug molecules, where the drug is being trapped and can easily survive some harmful factors in the biological system. A vasopressin derivative formulated in a vesicular delivery system demonstrated enhanced oral bioavailability, with superior drug stability and entrapment efficiency (Yoshida et al. 1992). This proniosomal formulation of insulin is one instance of peptide drug delivery. The presence of cholesterol and non-ionic surfactants stabilizes this drug from enzymatic degradation and other biological obstacles. This method provides the stability and significant bioavailability of insulin. Compared to traditional injectable forms, the proniosomal delivery system can provide a more practical and efficient way to manage controlled release and a higher absorption rate in the GI tract (Teaima et al. 2022).

### 7.5. Application in immune response study

Proniosomes have proven valuable for studying the immune response because of their immunological selectivity, low toxicity, and high stability. Researchers are examining the nature of the immune response triggered by antigens using proniosomes and niosomes (Chandra and Sharma 2008; Kakar 2010). A current study investigated a proniosomal formulation designed for malaria vaccine antigens. These findings indicated that this delivery method significantly increased the immune response, resulting in higher antibody levels in animal models compared to standard formulations (Shegokar 2023). On the other hand, researchers have examined the use of proniosomes to deliver CpG oligodeoxynucleotides as adjuvants. This approach improved the immune response to associated antigens, leading to a stronger T helper (Th1) cell immune reaction in experimental mice (Gogoi et al. 2018).

### 7.6. Application in hemoglobin delivery

Numerous carrier proteins are found in the blood. Hemoglobin can be easily transported to various locations in the biological systems through proniosomes once they are converted to niosomes. Owing to their permeability to oxygen, niosomal and proniosomal vesicles act as hemoglobin transporters (Radha et al. 2013). These vesicles are important candidate to improve hemoglobin's stability, bioavailability, and controlled release. Proniosomes protect hemoglobin from oxidative damage and facilitate targeted delivery to particular tissues, including ischemic regions with critical oxygen demand. They also allow for non-invasive delivery methods, which enhance patient comfort and compliance. Proniosomes, taken as a whole, offer a flexible and successful method for enhancing hemoglobin treatment in a range of therapeutic situations (Biju et al. 2006; Durga and Veera 2020).

### 7.7. Application in gene delivery

After moisture-induced conversion to niosomes, proniosomal systems promote efficient gene delivery. Puras et al. described a technique that uses niosomes to transfer the pCMSEGFP plasmid to the retina, highlighting their potential for ocular gene therapy (Puras et al. 2014). By utilizing the solvent emulsification-evaporation process, they developed the niosomes using the cationic lipids 2,3-di(teradecyloxy)propan-1-amine, aqualene, and polysorbate 80. These findings demonstrated that niosomes are capable of shielding DNA from enzymatic degradation and facilitating the entry of gene materials into cells (Puras et al. 2014).

**Figure 3.**
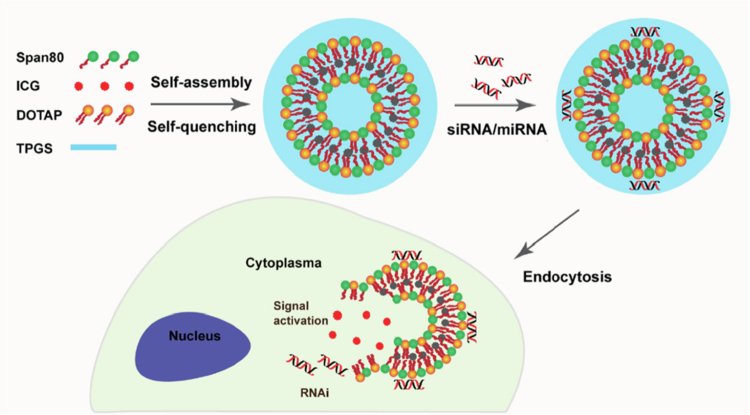
Schematic of vesicular self-assembly and subsequent endocytosis-mediated siRNA/miRNA delivery. The nanostructure facilitates targeted payload release through signal-triggered membrane fusion. Adapted with permission from reference (Teaima et al. 2022). Copyright © 2018 American Chemical Society.

According to a previous study, niosomes can be used as a vehicle to transfer RNAs to human mesenchymal stem cells in order to stimulate cell differentiation. The niosomes have unique characteristics that helps to deliver siRNA/miRNA into cells. These cationic niosomes were developed by using 1,2-dioleoyl-3-trimethylammonium propane (DOTAP), PEGylated lipid (TPGS), and Span 80. Specific gene sequencing in human mesenchymal stem cells (hMSCs) can occur when RNAs are complexed with niosomes at the appropriate ratio, and the surface charge is approximately 29.5 mV according to DLS measurements (Figure 3) (Yang et al. 2018).

### 7.8. Other applications

The engineered architecture of proniosomes enables precision drug targeting and controlled release kinetics, expanding their utility beyond conventional disease management.

***Sustained release***

The pharmaceutical field increasingly adopts proniosomal and niosomal vesicles as advanced platforms for sustained and on-demand release of drugs, addressing critical limitations of traditional drug formulations. After encapsulating a high payload of drug into the core of the vesicles, a minimized dissolution rate was achieved, along with sustained release action and an improved therapeutic index (Madan et al. 2016; Teng et al. 2019). Proniosomes have proven effective in providing prolonged release of various active pharmaceutical ingredients. For instance, a study demonstrated that paclitaxel-containing proniosomes can achieve sustained release, enhancing the drug's bioavailability and improving anticancer efficacy with greater therapeutic outcomes (Najlah et al. 2018). When nonsteroidal anti-inflammatory drugs (NSAIDs) were delivered transdermally, proniosomal formulations considerably prolonged drug release (71.32% over a period of 24 h) in comparison to conventional techniques (Kumar and Jain, 2016 ). An optimized lornoxicam proniosomal gel developed by Madan et al. achieved 66.98% EE and exhibited sustained drug release for more than 24 h (Madan et al. 2016). Proniosomes also maintain the dosing of drugs at a constant level over time, according to a study of metformin hydrochloride for type 2 diabetes treatment. The results highlight the potential of proniosomes to enhance drug distribution and improve therapeutic efficacy, with high drug encapsulation and controlled release of 75.90% after 24 h (Loona et al. 2012).

***Targeted drug delivery***

Proniosome-based drug delivery is a principal method to achieve local therapeutic action because of the smaller size of nanoparticles and high permeability through connective and epithelial tissues. Proniosomes demonstrated that when a drug is targeted locally, its potency and efficacy are enhanced while minimizing systemic toxicity. For instance, an anti-carcinogenic agent, gossypin-loaded proniosomal gel, demonstrated active transportation of the drug through skin layers for melanoma therapy. As a result, the drug has shown potential and less toxicity (Rajkumar et al. 2021). Another study revealed that an intranasal proniosomal powder loaded with artemether showed enhanced stability, superior penetration, prolonged drug release, and effective brain targeting for the treatment of cerebral malaria induced by *Plasmodium falciparum* (Uddin et al. 2024). In-vitro and ex-vivo studies have shown that buccal delivery of proniosomal-benzocaine provides targeted sustained release for prolonged anesthesia, making it highly valuable for managing mucosal pain (Abd El-Alim et al. 2014).

## 8. Biocompatibility and toxicological challenges

Proniosomes are considered to have a good safety profile because of the use of safe and biocompatible components in their preparation. However, some toxicities may occur owing to the chemical nature of surfactants (Yadav et al. 2011). In studies where drugs were delivered by oral and parenteral routes, proniosomes did not exhibit any harmful effects. For example, a lornoxicam-loaded proniosomal formulation was found to be safe and effective (Vijaya et al. 2018). Similarly, lomefloxacin proniosomes revealed that they had a good level of ocular tolerability and did not cause eye redness or inflammation (Khalil et al. 2017). In addition, 5-fluorouracil-loaded proniosomal gel demonstrated superior therapeutic efficacy and enhanced safety profiles in cancer treatment (Madan et al. 2016). Hence, collective evidence has established the high safety and excellent tolerability of proniosomes, underscoring their potential for safe and effective therapeutic applications.

## 9. An analysis of opportunities and obstacles of proniosomes

Proniosomes are positioned as a truly next-generation vesicular platform owing to their compelling profile. However, in order to direct rational composite engineering and clinical advancement, their limitations must be addressed. Among their major advantages, proniosomes significantly improve the solubility and permeability of poorly water-soluble drugs, leading to superior systemic absorption and therapeutic plasma concentrations (Ramkanth et al. 2018). Their inherent architecture enables the controlled and sustained release of active drug molecules to achieve long-term effects and minimize frequent dosing, which is ideal for managing chronic disease (Verma et al. 2016). Furthermore, a variety of therapeutic agents, ranging from small molecules to proteins and nucleic acids, can be effectively encapsulated in the versatile proniosomal matrix. These agents can be engineered for targeted delivery through surface modifications to maximize therapeutic efficacy while minimizing off-target adverse effects. They also reduce systemic exposure to free drugs by incorporating them into biocompatible vesicles, hence reducing the risk of organ-specific toxicity. From a practical standpoint, their exceptional dry state enhances their physicochemical stability by reducing the hydrolysis/oxidation risks associated with aqueous vesicular systems (Kumari et al. 2014). Notably, the methods employed for the preparation of proniosomes are simple and thus amenable to large-scale manufacturing.

**Figure 4.**
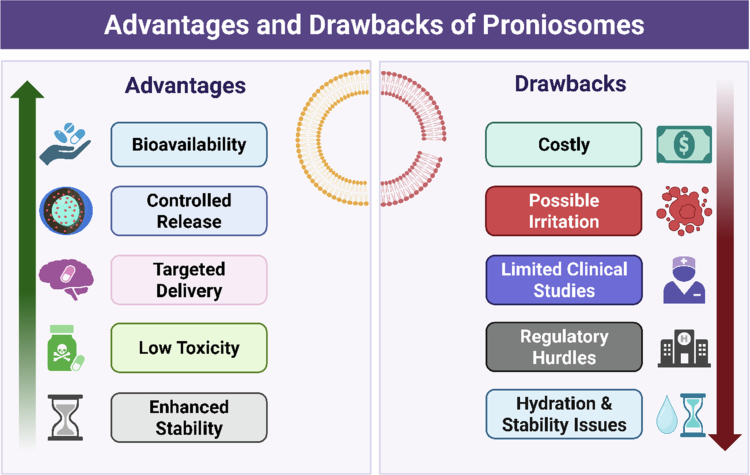
The benefits and challenges in proniosomal drug delivery. This image was created with BioRender (https://biorender.com/).

However, their transformative potential is offset by several translational obstacles. For instance, higher material (e.g. coating materials, surfactants, cholesterols, etc.) costs for the preparation of proniosomes could impair their industrial production. Surfactant-associated irritation remains a formulation challenge, as some non-ionic surfactants can perturb epithelial integrity at application sites. A substantial absence of comprehensive clinical investigations, along with the inherent complexity of securing regulatory approval for innovative delivery systems, can significantly delay market entry. Since hydration is the primary mechanism of action for proniosomes, their use may not always be practical in clinical settings. Although they are stable in their dry form, their stability may be compromised once hydrated (Bachhav 2016; Benetti et al. 2024). Therefore, a balanced examination of these issues, as illustrated in Figure 4, is critical for strategically directing the future development and clinical translation of proniosomal technology.

## 10. Navigating the translational pathway: from bench to bedside

The appealing preclinical data on proniosomes highlight their great promise; nonetheless, the path from intriguing laboratory discovery to mainstream clinical therapy is plagued with acknowledged obstacles that necessitate strategic advances. A thorough examination of this path is required to define the future of this technology.

### 10.1. Current hurdles and the commercial landscape

Proniosomes have considerable challenges in clinical translation, despite their potential for medicinal use. Widespread adoption is hampered by expensive materials (Khatoon et al. 2017), surfactant-induced irritation (Rajabalaya et al. 2016), and inadequate clinical validation. A comparative clinical study on a tretinoin-loaded proniosomal gel (N8G) for acne treatment in 12 Egyptian patients over the age of 18 years reported superior therapeutic efficacy and a remarkable reduction in skin irritation compared to a standard marketed product (Rahman et al. 2015). While a clear trend toward improvement was observed, the results were not statistically significant, potentially due to the limited statistical power of the small cohort. Similarly, a non-blind, two-treatment, two-period, randomized, crossover clinical investigation of sugar ester-based proniosomes for the transdermal delivery of vinpocetine confirmed enhanced drug permeation and established a well-tolerated profile in human subjects when compared to an oral commercial tablet (El-Laithy et al. 2011). These studies are small (*n* = 12), short-term (≤4 weeks), and use surrogate endpoints (lesion counts, plasma concentrations) instead of direct clinical outcomes. Additionally, a major absence of large-scale clinical trials data (e.g. on ClinicalTrials.gov) has stalled the transition of this system from the lab to the clinic. This translational gap not only leaves key concerns about human pharmacokinetics, safety, and efficacy unsolved, but also discourages large-scale pharmaceutical investment.

Furthermore, a review of the intellectual property landscape reveals a thriving, innovative field with significant commercial possibilities. As shown in Table 3, key patents demonstrate a strategic focus on novel niosomal and proniosomal formulations and their targeted therapeutic applications. Foundational, now-expired patents from industry leader L’Oréal pioneered noisome stabilization (EP0611207A1) (Alain Ribier 2001) and cosmeceutical application (US4830857A) (Handjani and Ribier, 1989 ) methods. Furthermore, recent filings (US10364350B2) (Nallani et al. 2019) reflect a shift from broad platform technologies, such as targeted vesicular constructs (US6306838B1) (Singh and Jain 2001) and pro-micelle compositions (US6951655B2) (Cho and Lee 2005), to specialized, high-value vesicular systems. Sustained innovation is evidenced by the predominance of active patents, with expirations extending to 2043. Despite active patenting, the portfolio is dominated by formulation/process improvements, with only a few late-stage clinical candidates, indicating a persistent translation gap. Commercialization becomes more difficult because of regulatory complications and obligatory hydration requirements (Bachhav 2016; Benetti et al. 2024). Additionally, formulation complications arise from stability issues after hydration. Realizing the full market potential of proniosomes requires addressing these constraints through robust manufacturing, tailored excipients, comprehensive characterizations, long-term stability studies, and stringent clinical testing.

**Table 3.** Key patents in proniosomal and niosomal drug delivery.

Patent number	Assignee	Innovation/Title	Status/Expiration	References
KR102334706B1	Korea Cosmetics Manufacturing Co Ltd.	Niosome composition comprising stabilized retinal and personal care composition containing the same	Granted-2021 (active)/Anticipated expiration-2041	Kang et al. (2021)
CN102579340A	Beijing Increase Pharmaceutical Research Institute Co Ltd., Beijing Increasepharm Co Ltd.	Sinomenine vesicle and preparation and preparation method thereof	Granted-2016 (active)/Anticipated expiration-2031	Hu Jie (2016)
US11944604B1	King Saud University	Nanoformulation of spriooxindole and methods for treating hepatocellular carcinoma	Granted-2024 (active)/Anticipated expiration-2043	Barakat et al. (2024)
CN103340823A	Southern Medical University	Formulation of paeonol proniosomes and preparing method thereof	Granted-2015 (active)/Anticipated expiration-2033	Qiang et al. (2015)
US4830857A	L’Oréal SA	Cosmetic and pharmaceutical compositions containing niosomes and a water-soluble polyamide, and a process for preparing these compositions	Granted-1989/Expired-2006	Handjani and Ribier, 1989
EP0611207A1	L’Oréal SA	Process for stabilization of vesicles of amphiphilic lipids and composition for topical application containing these stabilized vesicles	Granted-2001/Expired-2014	Alain Ribier (2001)
US6306838B1	Panacea Biotec Ltd.	Targeted vesicular constructs for cyto protection and treatment of *H. pylori* infections	Granted-2001/Expired-2020	Singh and Jain (2001)
US6951655B2	IMI Biomed Inc	Pro-micelle pharmaceutical compositions	Granted-2005/Expired-2022	Cho and Lee (2005)
US10364350B2	Agency for Science Technology and Research Singapore	Vesicular system and uses thereof	Granted-2019 (active)/Anticipated expiration-2030	Nallani et al. (2019)

### 10.2. Future roadmap: bridging the gap with emerging innovations

In the future, proniosomes can enlighten the pathway of long-acting drug delivery systems to cure diseases while minimizing the rate of side effects and enhancing the therapeutic index through targeted and controlled release to specific tissues and cells. Sustainability will also play a significant role, with a focus on biodegradable materials. Artificial intelligence (AI) will revolutionize proniosome engineering via machine learning-driven QbD. This could be demonstrated by the use of artificial neural networks, deep neural networks to predict optimal surfactant‒cholesterol‒polymer ratios, and Gaussian process models to forecast post-hydration vesicle size, which collectively automate the development of highly stable, effective, and personalized stimuli-responsive composite proniosomes with precise release kinetics (Zidan et al. 2011; Kashani-Asadi-Jafari et al. 2022; Dong et al. 2025). While research evolves, it will be imperative to create educational measures complemented by financial resources to provide professional training in this advanced technology, which will lead to its wider acceptance and views for regulatory endorsement. Collectively, these advantages position proniosomes as a transformative platform poised to redefine future drug delivery paradigms.

## 11. Conclusion

Proniosomes are an essential aspect of the drug delivery system and have evolved far beyond their origins as stable niosome precursors. They evolved from proliposomal ancestors to next-generation theranostic hybrids to address the inherent instability of conventional vesicular systems by improving their bioavailability, stability, and targeted drug delivery. This was discovered largely due to the advancement in research; and consequently, the combination of nanotechnology as well as various formulation methods are expected to result in enhanced functions, with a focus on bioactive drug molecules as well as biologicals. Furthermore, the proniosomal platform is highly flexible, which aligns with the current shift towards personalized therapeutics. This tunability is evident in its composite character, which can be enhanced by ligands and polymers, in addition to the precise control it provides over size and composition. Switching to biodegradable and sustainable materials, along with AI-integration in product development, will not only address environmental concerns but also promote the marketing of sustainable options. However, extensive clinical translation is hampered by a lack of robust human trials to validate preclinical proofs-of-concept, and the requirement to build standardized, QbD manufacturing methods to overcome regulatory constraints.

The possibility of using proniosomal composite systems in combination with other techniques of drug delivery offers good opportunities for enhancing the effectiveness and preventing adverse effects. Nevertheless, more promising research studies and trials, as well as the approval of various regulatory authorities, need to be conducted to enhance the clinical adoption of the proniosomes. As these concerns are dealt with, awareness about the proniosome technology is imperative for training aspiring researchers and members of the health sciences. In summary, ongoing research on proniosomes will be essential for harnessing their full potential in clinical settings. As we deepen our understanding of this technology, proniosomes may significantly transform drug delivery, ultimately benefiting patient outcomes and therapeutic strategies.

## Data Availability

Data sharing is not applicable to this article, as no new data were created or analyzed in this study. The datasets used for the present study were collected from journals such as Google Scholar, ScienceDirect, Web of Science, PubMed, SpringerLink, JSTOR, ResearchGate, etc.
